# PERILS, PITFALLS, AND POTENTIALS OF NETWORKING IN INTERPROFESSIONAL EDUCATION: TAKING ON THE CHALLENGES

**DOI:** 10.1093/geroni/igz038.1575

**Published:** 2019-11-08

**Authors:** Phillip Clark

**Affiliations:** University of Rhode Island, Kingston, Rhode Island, United States

## Abstract

Gerontology and geriatrics are conceptualized as quintessential interdisciplinary fields. To understand aging and provide quality care to older adults, you need an interdisciplinary perspective and an interprofessional team. However, academic and clinical settings—with their vertical structures—require bridging strategies to connect the disciplinary dots. Implicit in this approach is the need for creating networks to support interdisciplinary education in both classroom and experiential settings. Taking on these challenges requires emphasizing the importance of key competencies for both gerontology and interprofessional practice with older adults, including foundational, interactional, and contextual dimensions. These competencies recognize the unique perspectives, contributions, and roles of different disciplines, and create the connections critical for promoting and sustaining interprofessional education. Strategies for developing and maintaining interprofessional networks include: (1) identifying forces driving and restraining change, (2) matching strategies for promoting networks to readiness for system change, and (3) enlisting external forces to make and maintain changes.

